# Deep learning segmentation of major vessels in X-ray coronary angiography

**DOI:** 10.1038/s41598-019-53254-7

**Published:** 2019-11-15

**Authors:** Su Yang, Jihoon Kweon, Jae-Hyung Roh, Jae-Hwan Lee, Heejun Kang, Lae-Jeong Park, Dong Jun Kim, Hyeonkyeong Yang, Jaehee Hur, Do-Yoon Kang, Pil Hyung Lee, Jung-Min Ahn, Soo-Jin Kang, Duk-Woo Park, Seung-Whan Lee, Young-Hak Kim, Cheol Whan Lee, Seong-Wook Park, Seung-Jung Park

**Affiliations:** 1 Division of Cardiology, Department of Internal Medicine, Asan Medical Center, University of Ulsan College of Medicine, Seoul, Korea; 2 Biomedical Engineering Research Center, Asan Medical Center, Seoul, Korea; 3 Department of Cardiology in Internal Medicine, School of Medicine, Chungnam National University, Chungnam National University Hospital, Daejeon, Korea; 4 Department of Electronic Engineering, Gangneung-Wonju National University, Gangneung, Korea

**Keywords:** Cardiology, Interventional cardiology

## Abstract

X-ray coronary angiography is a primary imaging technique for diagnosing coronary diseases. Although quantitative coronary angiography (QCA) provides morphological information of coronary arteries with objective quantitative measures, considerable training is required to identify the target vessels and understand the tree structure of coronary arteries. Despite the use of computer-aided tools, such as the edge-detection method, manual correction is necessary for accurate segmentation of coronary vessels. In the present study, we proposed a robust method for major vessel segmentation using deep learning models with fully convolutional networks. When angiographic images of 3302 diseased major vessels from 2042 patients were tested, deep learning networks accurately identified and segmented the major vessels in X-ray coronary angiography. The average F1 score reached 0.917, and 93.7% of the images exhibited a high F1 score > 0.8. The most narrowed region at the stenosis was distinctly captured with high connectivity. Robust predictability was validated for the external dataset with different image characteristics. For major vessel segmentation, our approach demonstrated that prediction could be completed in real time with minimal image preprocessing. By applying deep learning segmentation, QCA analysis could be further automated, thereby facilitating the use of QCA-based diagnostic methods.

## Introduction

X-ray coronary angiography (CAG) is a primary imaging technique for diagnosing coronary diseases, one of the leading causes of death in the world. From CAG, the morphology of coronary arteries is obtained from real-time interpretation in the catheterization room, and quantitative coronary angiography (QCA) is used to provide objective quantitative measures. Over the past decade, QCA-based diagnostic methods have been introduced, such as the SYNTAX score for the evaluation of multi-vessel diseases^1^, angiography-derived fractional flow reserve (FFR)^2^, and prediction of plaque vulnerability^3^.

Because CAG is the projection of a three-dimensional (3-D) coronary artery onto a two-dimensional (2-D) plane, QCA is prone to image artifacts^4^. Overlaid blood vessels require considerable training for identifying target vessels and understanding coronary tree structures. Despite the use of computer-aided tools, such as the edge-detection method, manual correction is necessary for accurate segmentation of coronary vessels. Although novel image-processing methods have been proposed for automated detection of the entire vessel area^5–7^, the processing time required for applying multiple filters was not practical, and vessel identification was not considered. Recently, deep learning models have been introduced for CAG segmentation^8–12^. However, deep learning approaches for major vessel segmentation have not achieved prediction accuracy sufficient for clinical applications^10,11^.

In the present study, we proposed a robust method for major vessel segmentation using deep learning models, which was inspired by the integration of U-Net^13^ with deep convolutional networks^14,15^. Four deep learning models were evaluated using datasets from two institutes, and the impact of data composition and dataset size on segmentation performance was investigated.

## Methods

### Study population

**Figure 1:**
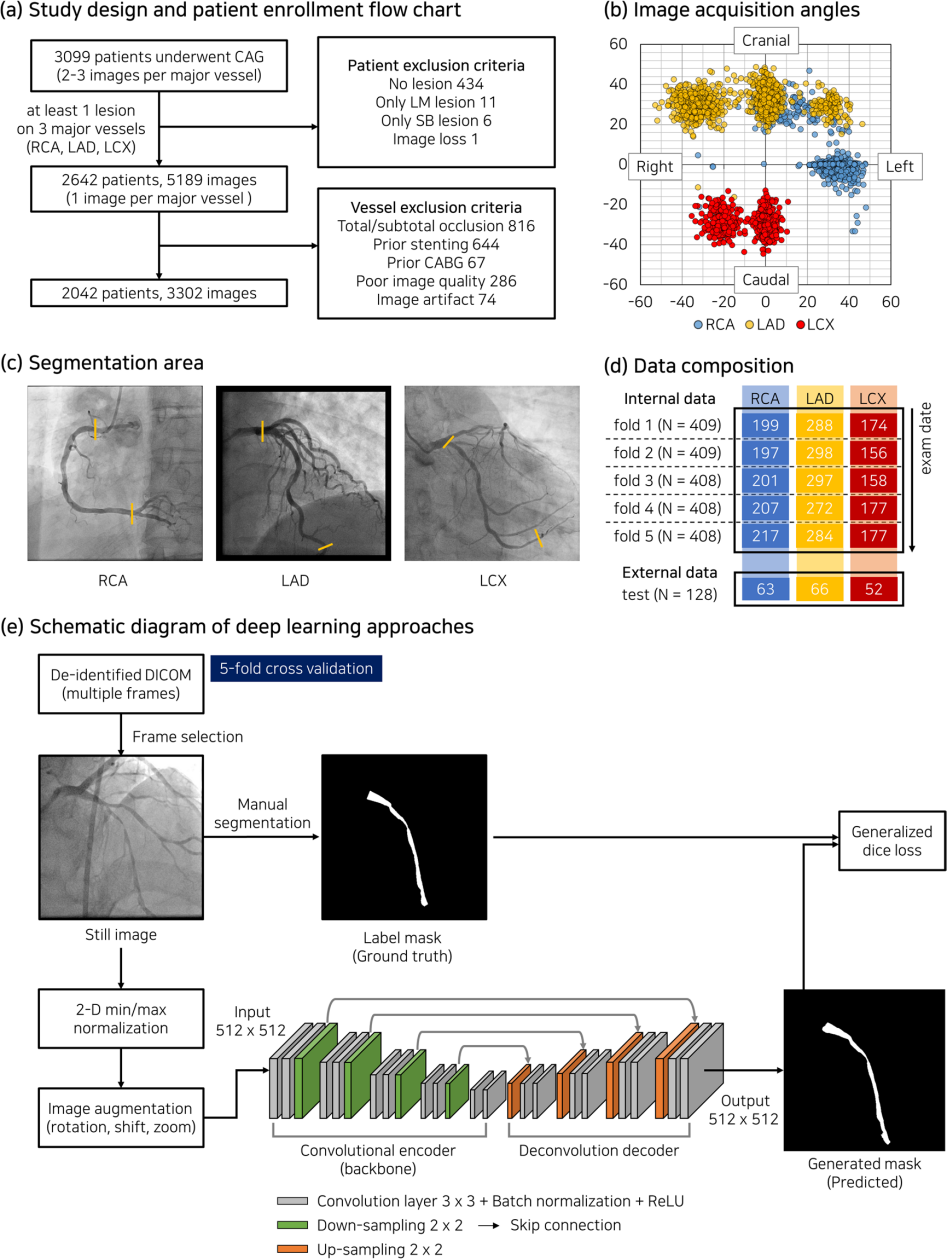
(**a**) Patient enrollment criteria. (**b**) Acquisition angles of X-ray coronary angiography (CAG). (**c**) Segmentation area of three major vessels, which bounded by yellow lines. (**d**) Number of patients (N) and vessel composition for internal and external datasets. (**e**) Schematic diagram of deep learning approaches using base architecture of U-Net model in this study. Each colored column in (**d**) indicates the number of images corresponding to major vessels.

In this study, 3309 patients who underwent X-ray coronary angiography in Asan Medical Center from Feb. 2016 to Nov. 2016 were retrospectively enrolled (Fig. 1a). This study complies with the Declaration of Helsinki and research approval was granted from the Institutional Review Board of the Asan Medical Center with a waiver of research consent. A series of X-ray coronary angiography comprised 2–3 acquisitions per three major vessels (right coronary artery, RCA; left anterior descending artery, LAD; left circumflex artery, LCX) at different acquisition angles (Fig. 1b). One image per major vessel with at least one lesion (diameter stenosis > 30%) was collected in projection, demonstrating the most severe narrowing. After excluding cases in which a coronary tree structure was not identified, such as coronary total chronic occlusion or overlaps of a medical device used for prior treatment, 3302 images of 2042 patients were ultimately included in this study. The baseline characteristics of the patients are summarized in Table 1.

**Table 1:** Patient summary.

	Internal (N = 2042)	External (N = 128)
Age (years)	64.3 ± 10.2	69.2 ± 10.3
Male, N (%)	1488 (72.9%)	87 (68.0%)
Diabetes mellitus, N (%)	658 (32.2%)	48 (37.5%)
Hypertension, N (%)	1213 (59.4%)	87 (68.0%)
Current smoker, N (%)	347 (17.0%)	22 (17.2%)
Hyperlipidemia, N (%)	403 (19.7%)	59 (46.1%)
Chronic renal failure, N (%)	120 (5.9%)	27 (21.1%)
Acute coronary syndrome, N (%)	250 (12.2%)	60 (46.9%)
**Number of diseased vessels, n (%)**		
Right coronary artery	1021 (30.9%)	63 (34.8%)
Left anterior descending artery	1439 (43.6%)	66 (36.5%)
Left circumflex artery	842 (25.5%)	52 (28.7%)
% Diameter stenosis (QCA)	46.6 ± 15.4	46.4 ± 15.2
Lesion length (mm)	18.2 ± 10.8	18.4 ± 11.2

N, number of patients; n, number of images; QCA, quantitative coronary angiography.

### X-ray coronary angiography and label process

Catheterization was performed through the femoral or radial routes using standard catheters, and coronary angiograms were digitally recorded. Personal patient information in the DICOM format was removed using the anonymization tool provided by the Health Innovation Big Data Center of Asan Medical Center. To generate label masks, a major vessel area on an angiogram was annotated by two experts with more than ten years of experience using CAAS workstation 7.5 (Pie Medical Imaging, Netherlands). On the image frame in the end-diastole phase, the initial mask of a major vessel boundary was generated using the semi-automatic edge-detection tool and then manually corrected. For each major vessel, the segmentation area was set from the ostium to the far distal (Fig. 1c). For RCA, the distal end of the segmented area was the bifurcation point between two branches—posterior descending artery (PDA) and posterolateral artery (PL). The capture and extraction of pixel information at the major vessel boundaries were performed using a customized Python script, and label masks were separately created (“internal dataset”; Fig. 1d).

### Networks

Four deep learning models were evaluated, which were constructed on the basis of U-Net architecture for semantic segmentation^13^ (Fig. 1e). Deep learning models based on U-Net have demonstrated powerful performance in binary semantic segmentation of grayscale images^13,15,16^. U-Net consists of a fully convolutional encoder called a backbone and a deconvolution-based decoder (‘SimpleUNet’). By replacing the backbone of U-Net with one of the most popular networks for image classification, such as ResNet101^17^, DenseNet121^18^, or InceptionResNet-v2^19^, deep learning models were applied for segmentation of X-ray angiography (see Appendix for network details). Input images of 512 × 512 pixels were normalized by using 2-D min/max normalization, and the initial weight was adopted from ImageNet for transfer learning.

### Loss function

Generalized dice loss (GD)^20^ was adopted to train the binary class segmentation. GD is defined as

$$GD=1-\frac{2{\sum }_{l}^{c}\,{w}_{l}{\sum }_{n}^{p}\,{G}_{ln}{P}_{ln}}{{\sum }_{l}^{c}\,{w}_{l}{\sum }_{n}^{p}\,({G}_{ln}+{P}_{ln})}),$$

where *c* is the number of classes, *p* is number of pixels, *G*_*ln*_ is the ground truth, and *P*_*ln*_ is the prediction result. The invariance term ${w}_{l}=1/({({\sum }_{n}^{p}({G}_{ln}/c))}^{2}+\varepsilon )$, which is inversely proportional to label frequency (${\sum }_{n}^{p}{G}_{ln}$), was introduced to mitigate the class imbalance between the major vessel region and other areas, where $\varepsilon ={10}^{-6}$. When *w*_*l*_ is equal between classes, GD is inherently the same as the dice loss. The major vessel area accounted for 2.69% ± 0.86% of 512 × 512 pixel images in the internal dataset.

### Training setup

Prediction models were trained for 400 epochs at maximum with a mini-batch size of 16. Data augmentation was performed with rotation (−20° to 20°), translation shift (0–10% of image size in horizontal and vertical axes), and zoom (0–10%). For training, an Adam optimizer^21^ was applied with β1 = 0.9 and β2 = 0.999, and the learning rate, which was initially set to 10^−3^, was reduced by half up to less than 10^–6^ each time the validation loss remained saturated for 20 epochs. The deep learning networks were implemented in Python using TensorFlow library and trained on a workstation with Intel i9-7900X CPU 3.3 GHz, 128 GB RAM, and four NVIDIA Geforce GTX 1080 Ti GPUs.

The evaluation metrics used to assess the predictability of the deep learning models were precision, recall, and F1 score, which were defined as precision = TP/(TP + FP), recall = TP/(TP + FN), and F1 = 2 × precision × recall/(precision + recall), where TP is true positive, FP is false positive, and FN is false negative. Evaluation metrics were calculated only for the major vessel area, i.e. TP represents the number of pixels for which the major vessel area was accurately predicted.

### Dataset and experimental setup

The constructed dataset was divided into five folds according to the exam date (Fig. 1d). Each fold had almost the same number of patients, which avoided the subdivision of the angiograms of a patient into different folds. First, to compare the segmentation performance of deep learning networks, cross validation was applied to each fold comprising three major vessels (Table 2). Then, to investigate the impact of the dataset composition, deep learning analyses were conducted with a separate dataset for each single major vessel, similar to the previous approaches^10,11^. In the cross validation, the fold proportion of training, validation, and test sets was 3:1:1, and the fold composition was changed in sequence under cyclic permutation. The impact of the dataset size on major vessel segmentation was also evaluated. The number of images in the training and validation sets was increased with increment of a fold, while fold 5 was fixed as a test set (Table 2).

**Table 2:** Summary of tests to evaluate the segmentation performance of deep learning networks.

Test name	Dataset			Major Vessel	Evaluation area
	Training	Validation	Test		
Hyperparameter	Fold 1–3	Fold 4	Fold 5	RCA + LAD + LCX	512 × 512
Combined dataset	Cross validation (Fold ratio = 3:1:1)			RCA + LAD + LCX	512 × 512 128 × 128^†^
Separate dataset	Cross validation (Fold ratio = 3:1:1)			RCA	512 × 512
				LAD	
				LCX	
Data size	Fold 1–4^*^ (Image ratio = 3:1)		Fold 5	RCA + LAD + LCX	512 × 512
External validation	Fold 1–3	Fold 4	External	RCA + LAD + LCX	512 × 512

RCA, right coronary artery; LAD, left anterior descending artery; LCX, left circumflex artery; ^†^The center of the evaluation area was set at the midpoint of the most narrowed location in a major vessel; ^*^Dataset size was gradually increased from fold 1 with increment of a fold.

### External validation

Although each major vessel has a standard view for CAG acquisition, the CAG characteristics are affected by clinical settings, such as view angle, magnification ratio, use of contrast media, and imaging system^22^. To evaluate the reliability and effectiveness of our prediction models, CAG images of 128 patients who visited Chungnam National University Hospital from Feb. 2016 to Nov. 2016 were collected and tested (Table 1). A total of 181 label masks in the “external dataset” was created using the identical protocol as the internal dataset. External dataset was used as the test set with the trained model using the internal dataset (Table 2). Research approval was granted from the Institutional Review Board of the Chungnam National University Hospital with a waiver of research consent.

### Statistical analysis

Continuous values are presented as mean ± standard deviation or median and interquartile range, as appropriate. Categorical variables are presented as numbers and percentages. The Mann-Whitney U test was applied to assess the differences in evaluation metrics associated with deep learning networks, dataset composition (combined vs. separate), and dataset size. The Kruskal-Wallis test was used to evaluate the impact of the minimum lumen diameter on the local F1 score around the stenosis. Values of p < 0.05 were considered statistically significant. Statistical analyses were performed using R package and SPSS 17.0 for Windows (SPSS, Inc., Chicago, IL, USA).

## Results

### Effects of hyperparameters on segmentation performance

To determine the hyperparameter set for evaluation, segmentation performance was examined with varying the epoch limit for plateau, augmentation parameters and optimizer (Table 3). 20 epochs for the plateau and the rotation angle of 20° exhibited the highest F1 score among the parameter combinations considered. Image flip offset the improvement effect of other augmentation techniques. Adam optimizer showed a higher average and larger standard deviation of the F1 score than stochastic gradient descent (SGD) and root mean square propagation (RMSprop) methods.

**Table 3:** Impact of hyperparameters on segmentation performance with DenseNet121.

Test name	Optimizer	Plateau	Augmentation				F1 score
			Rotation	Translation	Zoom	Flip	
Plateau	Adam	5	20	0.1	0.1	—	0.919 ± 0.084
	Adam	20	20	0.1	0.1	—	**0.923 ± 0.078**
	Adam	40	20	0.1	0.1	—	0.921 ± 0.088
Augmentation	Adam	20	—	—	—	—	0.910 ± 0.104
	Adam	20	10	0.1	0.1	—	0.916 ± 0.096
	Adam	20	30	0.1	0.1	—	0.920 ± 0.092
	Adam	20	20	0.1	0.1	O	0.910 ± 0.112
Optimizer	SGD	20	20	0.1	0.1	—	0.905 ± 0.092
	RMSprop	20	20	0.1	0.1	—	0.921 ± 0.091

SGD, stochastic gradient descent; RMSprop, root mean square propagation.

### Performance in combined dataset of three major vessels

In major vessel segmentation, ResNet101, DenseNet121, and InceptionResNet-v2 statistically outperformed SimpleUNet in terms of recall, precision, and F1 score (p < 0.001; Table 4). DenseNet121 achieved the highest F1 scores of 0.917 ± 0.103 in total and 0.940 ± 0.058 in the RCA subset, respectively. Although LCX segmentation exhibited a lower performance compared with the other major vessels, the average F1 score of LCX was ≥ 0.878 for all the considered networks except SimpleUNet.

**Table 4:** Comparison of segmentation performance between deep learning networks for combined dataset of three major vessels. The highest F1 score was shown in bold.

	SimpleUNet			ResNet101			DenseNet121			InceptionResNet-v2		
**Internal dataset**												
	Re	Pr	F1	Re	Pr	F1	Re	Pr	F1	Re	Pr	F1
Total (n = 3302)	0.869 ± 0.151	0.885 ± 0.125	0.871 ± 0.130	0.915 ± 0.117^*^	0.916 ± 0.106^*^	0.913 ± 0.108^*^	0.921 ± 0.112^*†§^	0.918 ± 0.103^*^	0.917 ± 0.103^*†^	0.915 ± 0.116^*^	0.920 ± 0.105^*†‡^	0.915 ± 0.107^*^
RCA (n = 1021)	0.915 ± 0.112	0.929 ± 0.076	0.918 ± 0.086	0.941 ± 0.084^*^	0.939 ± 0.068^*^	0.937 ± 0.071^*^	0.945 ± 0.071^*§^	0.940 ± 0.064^*^	0.940 ± 0.058^*^	0.943 ± 0.072^*^	0.942 ± 0.063^*^	0.940 ± 0.058^*^
LAD (n = 1439)	0.872 ± 0.141	0.882 ± 0.114	0.872 ± 0.119	0.921 ± 0.104^*^	0.917 ± 0.090^*^	0.916 ± 0.094^*^	0.928 ± 0.094^*†§^	0.920 ± 0.085^*^	0.922 ± 0.084^*^	0.919 ± 0.105^*^	0.922 ± 0.094^*†‡^	0.918 ± 0.095^*^
LCX (n = 842)	0.809 ± 0.186	0.837 ± 0.166	0.813 ± 0.166	0.875 ± 0.156^*^	0.887 ± 0.151^*^	0.878 ± 0.150^*^	0.878 ± 0.158^*^	0.888 ± 0.150^*^	0.879 ± 0.153^*^	0.875 ± 0.158^*^	0.890 ± 0.148^*^	0.879 ± 0.151^*^
**External dataset**												
	Re	Pr	F1	Re	Pr	F1	Re	Pr	F1	Re	Pr	F1
Total (n = 181)	0.764 ± 0.251	0.855 ± 0.202	0.791 ± 0.226	0.846 ± 0.232^*^	0.871 ± 0.191	0.849 ± 0.214^*^	0.898 ± 0.155^*^	0.904 ± 0.126^*^	0.896 ± 0.138^*^	0.887 ± 0.172^*^	0.905 ± 0.144^*†^	0.890 ± 0.161^*†^
RCA (n = 63)	0.876 ± 0.172	0.934 ± 0.051	0.891 ± 0.126	0.918 ± 0.135	0.921 ± 0.075	0.911 ± 0.112	0.931 ± 0.096^*^	0.925 ± 0.062	0.924 ± 0.066	0.939 ± 0.072^*^	0.928 ± 0.073	0.930 ± 0.058^*^
LAD (n = 66)	0.788 ± 0.205	0.855 ± 0.173	0.814 ± 0.183	0.882 ± 0.185^*^	0.876 ± 0.179	0.878 ± 0.179^*^	0.914 ± 0.127^*^	0.901 ± 0.126	0.906 ± 0.122^*^	0.887 ± 0.146^*^	0.915 ± 0.090^*†‡^	0.892 ± 0.140^*^
LCX (n = 52)	0.599 ± 0.299	0.758 ± 0.292	0.638 ± 0.284	0.714 ± 0.314^*^	0.805 ± 0.271	0.738 ± 0.294^*^	0.836 ± 0.218^*†^	0.884 ± 0.174^*^	0.848 ± 0.197^*†^	0.825 ± 0.252^*†^	0.864 ± 0.232^*^	0.837 ± 0.239^*†^

Re, recall; Pr, precision; F1, F1 score; RCA, right coronary artery; LAD, left anterior descending artery; LCX, left circumflex artery. *, †, ‡ and § denote p < 0.05 versus the corresponding metrics of SimpleUNet, ResNet101, DenseNet121 and InceptionResNet-v2, respectively. p < 0.001 for all the evaluation metrics in the internal dataset when comparing SimpleUNet and one of the other deep networks.

**Figure 2:**
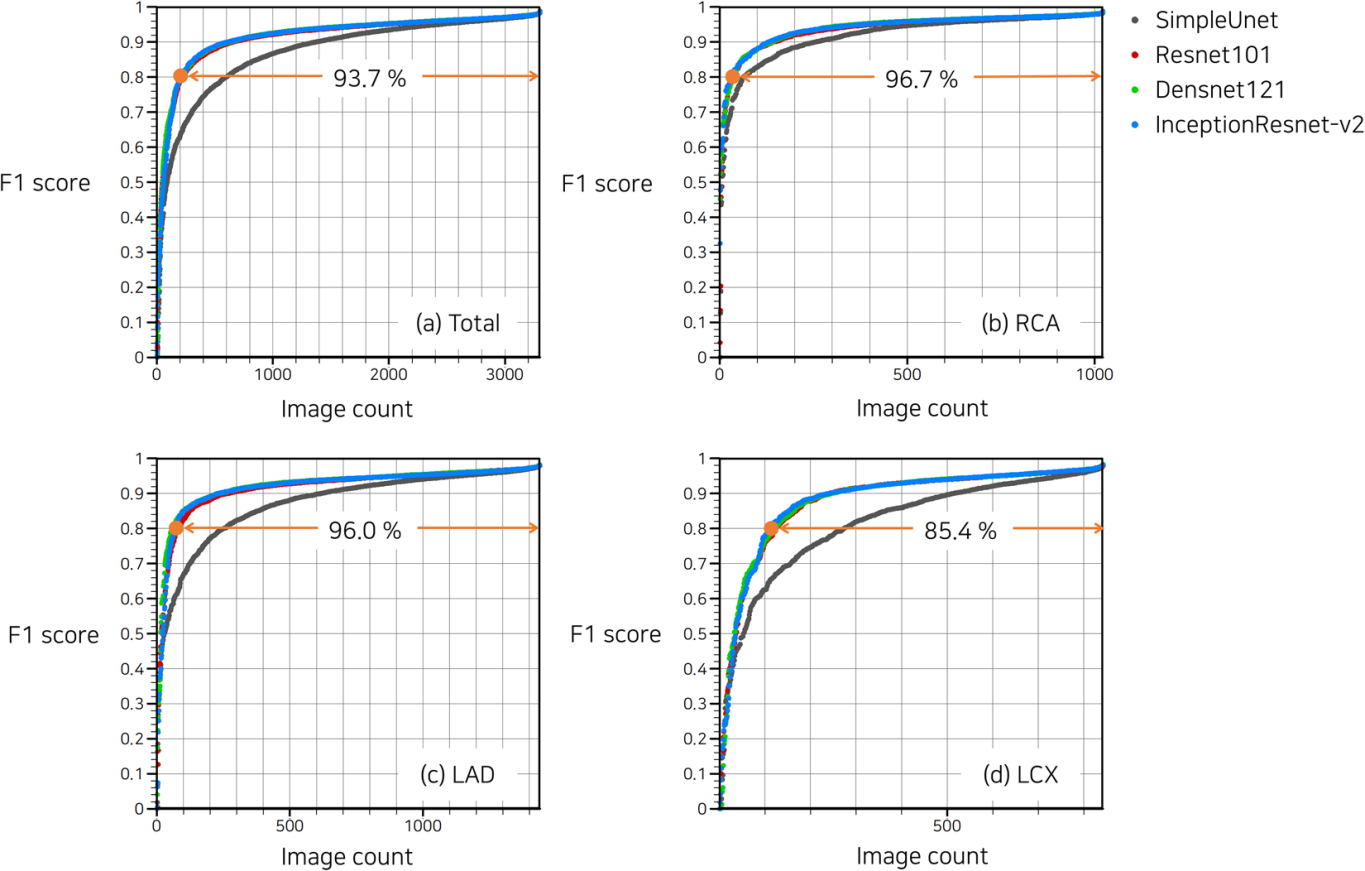
Cumulative histogram of F1 score in combined dataset of three major vessels. Proportion of images with F1 score > 0.8 predicted using DenseNet121 is indicated by the orange line.

In a cumulative histogram, 93.7% of the total images exhibited F1 score > 0.8 with DenseNet121 (Fig. 2a). Histogram differences between DenseNet121 and InceptionResNet-v2 were negligible for all three major vessels, especially for images of F1 score > 0.9 (Fig. 2b–d). Only for RCA, SimpleUNet provided outcome quality comparable with the other networks with deeper layers (Fig. 2b).

**Figure 3:**
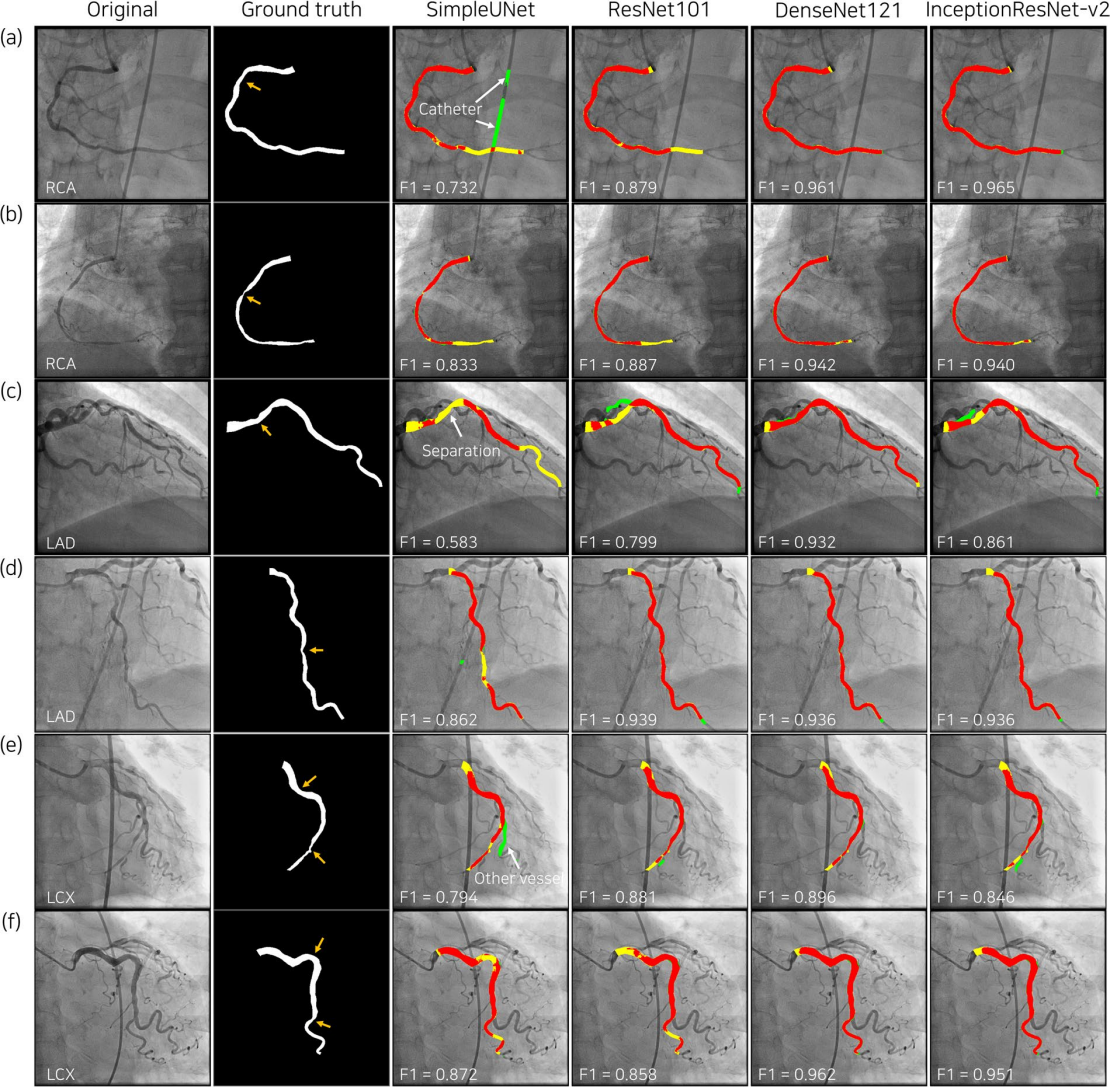
Representative results of major vessel segmentation. In the third to sixth columns, the predicted major vessel areas compared to the ground truth (second column) are indicated in red (true positive), yellow (false negative) and green (false positive). Orange arrows in the second column indicate coronary lesions.

**Figure 4:**
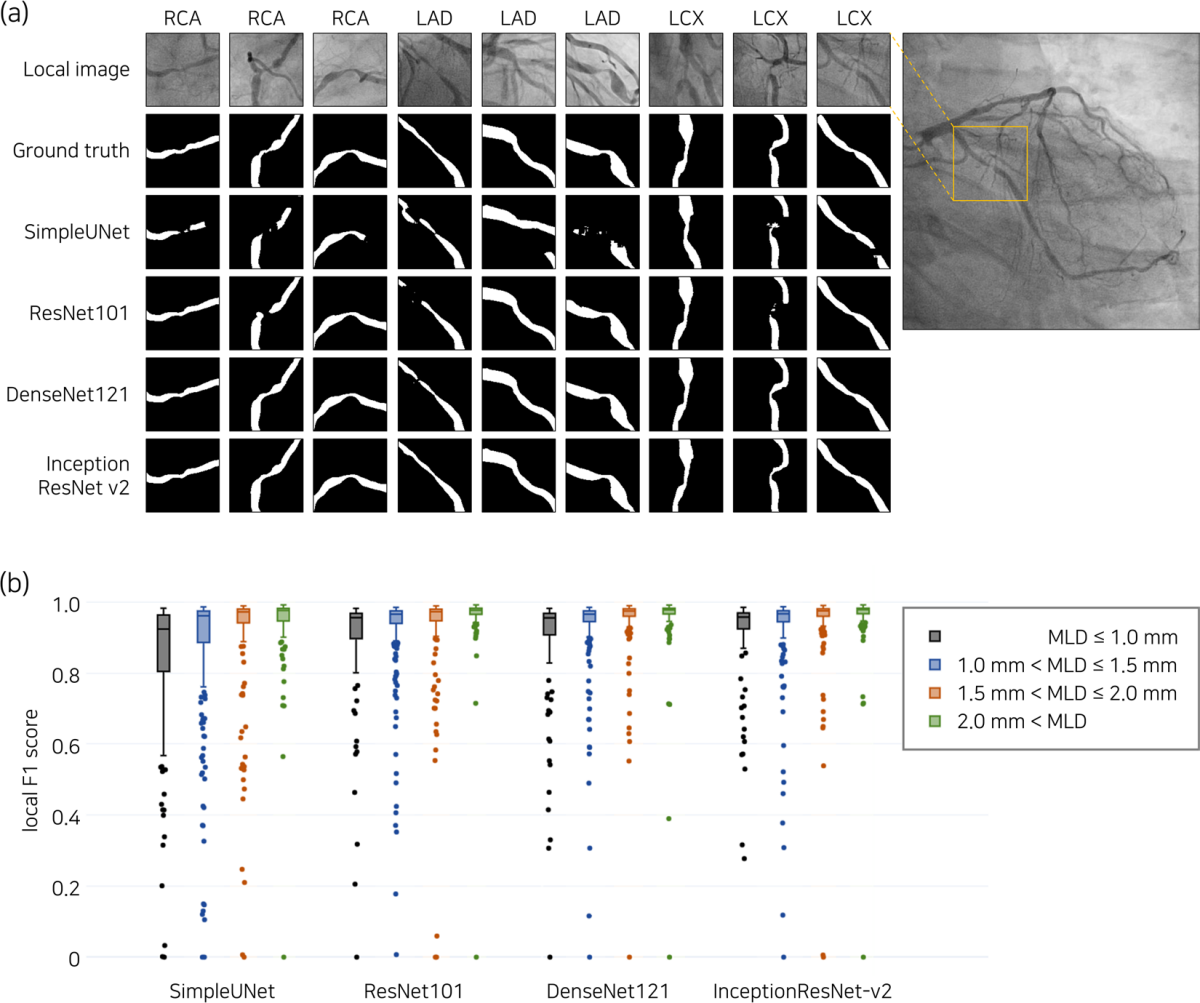
Representative examples of major vessel segmentation in the bounding box of 128 × 128 pixels around the stenosis are shown in (**a**). For fold 5, local F1 scores are compared among the four groups divided by the minimum lumen diameter (MLD) of the stenosis in (**b**), which results in p < 0.001 for all deep networks.

The representative examples of deep-learning segmentation are depicted in Fig. 3. Despite overlap with catheters and other blood vessels, DenseNet121 and InceptionResNet-v2 accurately predicted the lumen area in major vessels and exhibited improved connectivity at the site of stenosis. Around the stenosis, the lumen boundary at the most narrowed location was sharply captured by DenseNet121 and InceptionResNet-v2 (Fig. 4a).

### Analysis of segmentation errors in combined dataset

**Figure 5:**
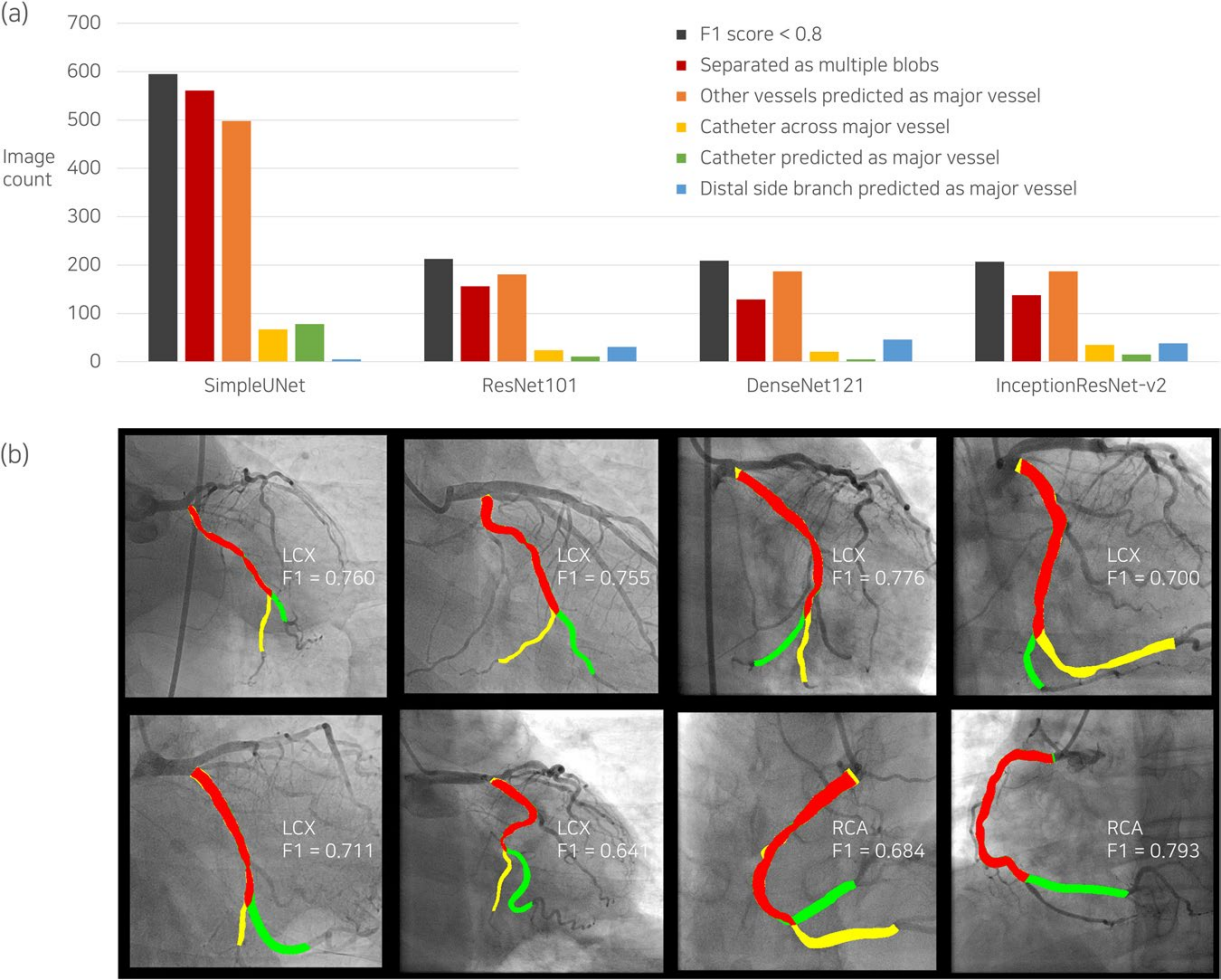
Error analysis of predicted major vessel area with F1 score < 0.8. The number of images corresponding to each error type is presented in (**a**), including cases with catheter across the major vessel as a reference. The cases in which large side branches misled the deep learning algorithms in the decision of the distal part of the major vessel, which may differ depending on the analyzer, are separately counted (blue bar in (**a**)), and the relevant examples are shown in the corresponding columns in (**b**). LCX, left circumflex artery; RCA, right coronary artery; F1, F1 score.

Error types of major vessel segmentation were classified for the images of F1 score < 0.8 (Fig. 5a). The most frequent patterns of segmentation errors were mask separation consisting of multiple blobs and misidentification. Catheters predicted as major vessels that hindered the improvement of segmentation performance^6^ were rarely found in the deep learning segmentation using DenseNet121. Among the images of low F1 scores, deep learning algorithms recognized a side branch as the distal part of major vessels, which may be an accurate identification, depending on the analyzers (Fig. 5b). In the local region around the stenosis, the minimum lumen diameter had a significant impact on the local F1 score (Fig. 4b).

### Comparison of separate and combined datasets

**Figure 6:**
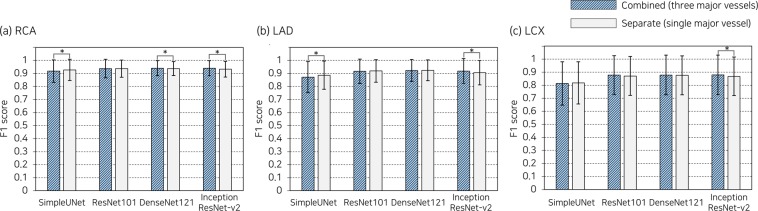
Comparison of segmentation performance between combined and separate datasets. p < 0.05 is denoted by an asterisk. RCA, right coronary artery; LAD, left anterior descending artery; LCX, left circumflex artery.

Separate datasets exhibited average F1 scores comparable to the combined dataset, despite learning with a smaller number of images (Fig. 6). Adding images of the other major vessels to the training set of a major vessel statistically improved the predictability of InceptionResNet-v2 (0.008–0.012 in F1 score), whereas SimpleUNet produced better outcomes with separate datasets of RCA and LAD (0.009–0.014 in F1 score).

### Impact of dataset size

**Figure 7:**
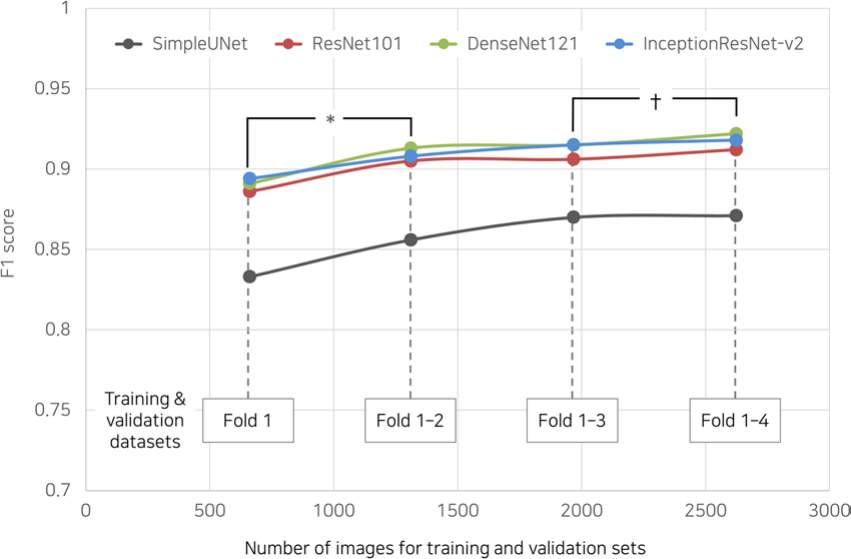
Impact of dataset size on segmentation performance of deep learning networks. *p < 0.001 for all deep networks; ^†^p = 0.027 for DenseNet121.

Even with approximately a quarter of the dataset, which was used for the cross validation (fold 1 in Fig. 7), the F1 score was 0.833 ± 0.142 for SimpleUNet, approaching an average of 0.9 for the other networks. When more than 3 folds were used for the training and validation sets, the segmentation capability was almost saturated.

### External validation

DenseNet121 demonstrated robustness to changes in the characteristic of angiographic images, achieving an F1 score of 0.896 ± 0.138. A noticeable degradation of LCX led to an overall reduction in the segmentation capability in the external dataset.

## Discussion

The major finding of the present study is that deep learning networks accurately identified and segmented the major vessels in X-ray coronary angiography. The average F1 score reached 0.917, and 93.7% of the images exhibited a high F1 score > 0.8. The most narrowed region at the stenosis was distinctly captured with high connectivity. Deep learning segmentation was assessed for a large number of angiographic images, and robust predictability was validated for the external dataset with different image characteristics.

In recent years, the combination of novel deep learning networks and U-Net^13^ has been proposed, remarkably improving the performance of semantic image segmentation^14^. DenseNet121 and InceptionResNet-v2 in the present study also demonstrated distinguished results for major vessel segmentation compared with the base model of U-Net, even with a relatively small dataset. DenseNet121 and InceptionResNet-v2 showed better results than the updated deep learning networks for multi-class segmentation^23,24^ in the current setting (see Appendix for comparison results). DenseNet121 tended to cover most of the lumen area (higher recall), whereas InceptionResNet-v2, with a similar F1 score, exhibited a propensity for excluding the area outside the lumen (higher precision). For DenseNet121, although the advantage of fewer parameters was offset with an increase in the memory usage of the inter-layer connection, the training time per epoch was 10.9% less than that for InceptionResNet-v2 (Table 5).

**Table 5:** Characteristics of deep learning networks in terms of training parameter and time. Training time and trained epoch were averaged from the results of cross validation for the combined set.

Network	Number of parameters	Training time (s)	Trained epoch	Training time per epoch (s)
SimpleUNet	7,762,914	41,959	324.8	129.18
ResNet101	51,605,611	39,994	298.8	133.85
DenseNet121	12,145,122	36,236	290.4	124.78
InceptionResNet-v2	62,061,698	37,535	268.0	140.06

The advantage of the current architecture for major vessel segmentation was that image preprocessing steps were minimized as min/max intensity normalization, which was seamlessly integrated into the deep learning model. The processes of extracting the entire coronary tree^5^ and segmenting catheters^6^ were not required. With the reduction in steps, the segmentation time was shortened to approximately 0.04 s per angiogram, which was shorter than the frame time of the recording system of 8–16 frame/s.

In comparison with the previous deep learning approaches using fully convolutional networks, higher F1 scores were achieved in the present study for RCA (0.704 in Au *et al*.^10^), LAD (0.676 in Jo *et al*.^11^), and three major vessels including normal arteries (0.890 in Jun *et al*.^12^). The primary reasons for improved segmentation performance in the current setting were the annotation criteria based on apparent anatomical landmarks and the application of appropriate techniques for image augmentation. By using the vessel ostium and major bifurcations as fiducial points, the inter- and intra-observer variability resulting from eye estimation to determine a vessel segment or a diseased lesion^25^ could be avoided. Near the bifurcation of LAD and LCX where overlapping lumen areas inevitably occurred, the ostium boundaries of the major vessels were consistently divided by referring to the adjacent image frames. Because the acquisition conditions of angiography vary within a certain range with respect to the standard imaging parameters (Fig. 1b), the limited amplitudes of the augmentation parameters were applied to rotation, translation, and zoom. Flip and crop techniques, which do not correspond to coronary anatomy and typical imaging setting, were excluded (Table 3). Although a single static image was used as an input to deep learning networks in this study, multi-view approaches could further improve the outcome by encompassing dynamic changes of the coronary arteries caused by heartbeat^26,27^.

Employment of deep learning applications could lead to changes in clinical activity based on the segmentation of X-ray angiography. Offline QCA analysis could be completed with less manual correction, and morphological information of the coronary lesion would be obtained by simply adjusting the reference locations. Therefore, the time required to calculate the SYNTAX score, which requires QCA analysis of entire coronary trees, would be reduced. Diagnostic methods with 3-D QCA^28^, which is constructed by combining the 2-D QCA of multiple angiograms could be further utilized. In the prediction of post-stenotic FFR and vulnerable plaque using fluid dynamics simulation^3^, the precise 3D reconstruction of a coronary artery is a prerequisite for accurate analysis^29^. The reduced time requirement for QCA analysis may allow for real-time application in the catheterization room, where clinician hands are not free to operate, eventually reducing the dependence of visual assessment and quantifiably guiding stent selection and optimization.

Although the deep-learning segmentation distinguished most luminal areas of the major vessels, there are some aspects to be improved for practical use. First, angiographic images with a low F1 score due to misidentification or separation may require greater modification of lumen boundaries compared with when edge-detection methods are applied. For a comprehensive interpretation of the coronary tree with stenoses, geometric analyses of the left main artery and side branches are necessary. In the assessment of bifurcation lesions, the evaluation of the location and shape of the narrowed area is important for both major and side branch vessels, which have different implications for cardiovascular risk. Among the excluded cases, segmentation of normal arteries and totally or subtotally occluded lesions may be necessary for more general use of the automated QCA, as well as the calculation of the SYNTAX score. Another limitation is that the tasks for QCA analysis before and after major vessel segmentation still rely on the competence of the analyst. To provide more automated and improved outcomes of QCA analysis with reduced analyst dependency, integration with conventional image processing techniques, such as edge detection and ECG-based frame selection, would be helpful in a complementary manner, as well as deep learning application across multiple stages. The size of the external dataset was small to generalize the segmentation capability of our method. Therefore, the robustness and reliability of the deep learning segmentation must be validated against the diversity of angiography characteristics, which vary depending on the institute or operator.

## Conclusion

Deep learning networks accurately identified and segmented the major vessels in X-ray coronary angiography. The prediction process could be completed in real time with minimal image preprocessing. By applying deep learning segmentation, QCA analysis could be further automated and, thus facilitating the use of QCA-based diagnostic methods.

## Supplementary Information

Supplementary information

## Data Availability

The datasets generated and/or analyzed during the current study are not publicly available because permission of sharing patient data was not granted by the Institutional Review Board but are available from the corresponding author on reasonable request.
