# Supplementary material for: Deep learning segmentation of major vessels in X-ray coronary angiography
Source: Sci Rep. 2019 Nov 15;9:16897. doi: 10.1038/s41598-019-53254-7 (PMC6858336; doi:10.1038/s41598-019-53254-7)
Supplement: Supplementary file 1 — Supplementary information [file 41598_2019_53254_MOESM1_ESM.pdf]

Table 1. Model architectures. Blue and green denote the encoder and decoder parts of deep learning architectures, respectively. (Repository: https://github.com/suyang93/Major-vessel-segmentation-on-X-ray-Angiography)

<table>
<tr><th>SimpleUNet</th><th>ResNet101</th><th>DenseNet121</th><th>InceptionResNet-v2</th></tr>
<tr><td colspan="4">Input shape = (512, 512, 3)</td></tr>
<tr><td>Conv3, 32
Conv3, 32
(U-Conv1)</td><td>Conv7-A, 64, stride 2
(R-Conv1)</td><td>Conv7-A, 64, stride 2
(D-Conv1)</td><td>Stem block, 64
(I-Conv1)</td></tr>
<tr><td>MaxPool2, stride 2</td><td>MaxPool3, stride 2</td><td>MaxPool3, stride 2</td><td>Stem block, 192
(I-Conv2)</td></tr>
<tr><td>Conv3, 64
Conv3, 64
(U-Conv2)</td><td rowspan="2">$3 \times \begin{bmatrix} 1 \times 1, & 64 \\ 3 \times 3, & 64 \\ 1 \times 1, & 256 \end{bmatrix}$
(R-Conv2)</td><td>6 × Dense block
Bottleneck, 128
(D-Conv2)</td><td>Inception block-A, 320</td></tr>
<tr><td>MaxPool2, stride 2</td><td>AvgPool2,
stride 2</td><td>10 × InceptionResNet-A, 320
(I-Conv3)</td></tr>
<tr><td>Conv3, 128
Conv3, 128
(U-Conv3)</td><td rowspan="2">$4 \times \begin{bmatrix} 1 \times 1, & 128 \\ 3 \times 3, & 128 \\ 1 \times 1, & 512 \end{bmatrix}$
(R-Conv3)</td><td>12 × Dense block
Bottleneck, 256
(D-Conv3)</td><td>Reduction-A, 1088</td></tr>
<tr><td>MaxPool2, stride 2</td><td>AvgPool2,
stride 2</td><td>20 × InceptionResNet-B, 1088
(I-Conv4)</td></tr>
<tr><td>Conv3, 256
Conv3, 256
(U-Conv4)</td><td rowspan="2">$23 \times \begin{bmatrix} 1 \times 1, & 256 \\ 3 \times 3, & 256 \\ 1 \times 1, & 1024 \end{bmatrix}$
(R-Conv4)</td><td>24 × Dense block
Bottleneck, 512
(D-Conv4)</td><td>Reduction-B, 2080</td></tr>
<tr><td>MaxPool2, stride 2</td><td>AvgPool2,
stride 2</td><td>10 × InceptionResNet-C, 2080</td></tr>
<tr><td>Conv3, 512
Conv3, 512</td><td>$3 \times \begin{bmatrix} 1 \times 1, & 512 \\ 3 \times 3, & 512 \\ 1 \times 1, & 2048 \end{bmatrix}$</td><td>16 × Dense block
Bottleneck, 1024</td><td>Conv1, 1536</td></tr>
<tr><td colspan="4">Upsamp2, stride 2 (Up4)</td></tr>
<tr><td>Concat
[U-Conv4, Up4]</td><td>Concat
[R-Conv4, Up4]</td><td>Concat
[D-Conv4, Up4]</td><td>Concat
[I-Conv4, Up4]</td></tr>
<tr><td colspan="4">Conv3, 256
Conv3, 256</td></tr>
<tr><td colspan="4">Upsamp2, stride 2 (Up3)</td></tr>
<tr><td>Concat
[U-Conv3, Up3]</td><td>Concat
[R-Conv3, Up3]</td><td>Concat
[D-Conv3, Up3]</td><td>Concat
[I-Conv3, Up3]</td></tr>
<tr><td colspan="4">Conv3, 128
Conv3, 128</td></tr>
<tr><td colspan="4">Upsamp2, stride 2 (Up2)</td></tr>
<tr><td>Concat
[U-Conv2, Up2]</td><td>Concat
[R-Conv2, Up2]</td><td>Concat
[D-Conv2, Up2]</td><td>Concat
[I-Conv2, Up2]</td></tr>
<tr><td colspan="4">Conv3, 64
Conv3, 64</td></tr>
<tr><td colspan="4">Upsamp2, stride 2 (Up1)</td></tr>
<tr><td>Concat
[U-Conv1, Up1]</td><td>Concat
[R-Conv1, Up1]</td><td>Concat
[D-Conv1, Up1]</td><td>Concat
[I-Conv1, Up1]</td></tr>
<tr><td>Conv3, 32
Conv3, 2</td><td colspan="3">Conv3, 32
Conv3, 32</td></tr>
<tr><td></td><td colspan="3">Upsamp2, stride 2</td></tr>
<tr><td></td><td colspan="3">Conv3, 16
Conv3, 16</td></tr>
<tr><td></td><td colspan="3">Conv3, 2</td></tr>
</table>

Conv3, 3 × 3 convolution layer; Conv7, 7 × 7 convolution layer; Conv1, 1 × 1 convolution layer; MaxPool2, 2 × 2 max pooling layer; MaxPool3, 3 × 3 max pooling layer; AvgPool2, 2 × 2 average pooling layer; Upsamp2, 2 × 2 up-sampling layer; Concat, concatenation layer.

Table 2 Comparison of major vessel segmentation between deep learning networks for fold 5. The evaluation was performed with the same experiment setting as the ‘Hyperparmeter’ test in Table 2.

	Recall	Precision	F1 score
SimpleUNet	0.872 ± 0.148	0.886 ± 0.118	0.873 ± 0.126
ResNet101	0.919 ± 0.106	0.916 ± 0.091	0.915 ± 0.092
DenseNet121	**0.933 ± 0.085**	0.916 ± 0.084	0.923 ± 0.078
InceptionResNet-v2	0.928 ± 0.095	**0.924 ± 0.092**	**0.925 ± 0.088**
PSPNet[1]	0.914 ± 0.098	0.906 ± 0.093	0.908 ± 0.088
DeepLab v3+[2]	0.914 ± 0.109	0.913 ± 0.098	0.911 ± 0.097

1. PSPNet: Pyramid Scene Parsing Network
    a. https://github.com/Vladkryvoruchko/PSPNet-Keras-tensorflow/blob/master/pspnet.py
2. DeepLab v3+: Encoder-Decoder with Atrous Separable Convolution for Semantic Image Segmentation
    a. https://github.com/Golbstein/Keras-segmentation-deeplab-v3.1/blob/master/deeplabv3p.py
